# The Hospital for Diseases of the Skin, Blackfriars

**Published:** 1909-02-06

**Authors:** 


### The Hospital for Diseases of the Skin, Blackfriars.

We had occasion to comment on the management of this Institution nearly two years ago (The Hospital, April 20, 1907, p. 75), and it would seem from recent events that the attention of the profession and of the funds and committees which watch over medical charities in London is again required in connection with it. We pointed out on the occasion referred to that the regulations of the hospital are so ill drawn that they are quite nugatory, and that a grave scandal had arisen through the manner in which the assistant surgeon with some aiders and abettors had raided the committee room, constituted themselves (without any authority or *locus standi*) a committee of Governors, and transacted business in a most irregular manner. Since that time the senior surgeons have both resigned in ineffective protest against these proceedings, and the entire medical staff of the hospital has consisted of the former assistant surgeon, to whom reference has already been made. This medical man died, as we are informed, on Monday, January 25, and a clinical assistant remains the only qualified practitioner who is in attendance to see patients. The situation is further complicated by the desire of the three trustees to lay down their administration of the trust funds, which amount to some seven or eight thousand pounds; by the fact that Messrs. Cooper, the auditors, have also declined, after forty years' service, further to be identified with the affairs of the hospital; and by the refusal of grants from the King Edward's and the Hospital Sunday Funds.

On the share of the recently deceased surgeon in the responsibility for this deplorable state of affairs, this is not the time to comment. Better is it to regard the future than the past, and to endeavour to find a solution for the difficult situation now existing. The trustees, who are anxious to give up their distinctly embarrassing position, want to transfer their trust to some body which will command public and professional confidence. Their feelings in regard to the question are easy to understand and to sympathise with; the problem is how best for the sake of the hospital and the poor population of the neighbourhood to replace them. One suggestion is that King Edward's Hospital Fund, or the Metropolitan Hospital Sunday Fund should for a time take over and administer the accumulated funds of the charity, appointing a committee or a board of Governors to conduct the affairs of the hospital until such time as a board which will command public confidence under some amended scheme of regulations can be built up from amongst the subscribers.

Legal advice would, of course, be necessary as to the exact manner by which such a transference from the present trustees can be brought about. It is at least quite plain that something must be done, and the sooner the better. How things have been allowed to drift into the present extraordinary condition may perhaps be profitably the subject of inquiry later, if merely to learn how best such occurrences may be avoided for the future. For the present the imperative need is for action, and that by some body or association of persons which will command the confidence of the public.

